# Accelerometer-Derived Sedentary and Physical Activity Time in Overweight/Obese Adults with Type 2 Diabetes: Cross-Sectional Associations with Cardiometabolic Biomarkers

**DOI:** 10.1371/journal.pone.0119140

**Published:** 2015-03-16

**Authors:** Genevieve N. Healy, Elisabeth A. H. Winkler, Charlotte L. Brakenridge, Marina M. Reeves, Elizabeth G. Eakin

**Affiliations:** 1 The University of Queensland, School of Public Health, Brisbane, Australia; 2 Baker IDI Heart and Diabetes Institute, Melbourne, Victoria, Australia; 3 Curtin University, School of Physiotherapy, Perth, Western Australia, Australia; Faculty of Biology, SPAIN

## Abstract

**Objective:**

To examine the associations of sedentary time and physical activity with biomarkers of cardiometabolic health, including the potential collective impact of shifting mean time use from less- to more-active behaviours (cross-sectionally, using isotemporal substitution), in adults with type 2 diabetes.

**Methods:**

Participants with overweight/obese body mass index (BMI; ≥25 kg/m^2^) (n = 279; 158 men, mean [SD] age = 58.2 [8.6] years) wore Actigraph GT1M accelerometers (waking hours; seven days) to assess moderate- to vigorous-intensity physical activity (MVPA), light-intensity activity, and sedentary time (segregated into non-prolonged [accumulated in bouts <30min] and prolonged [accumulated in bouts ≥30 min]). Cross-sectional associations with waist circumference, BMI, fasting blood (HbA_1c_, glucose, triacylglycerols, high-density lipoprotein cholesterol), and blood pressure of these activity variables (30 min/day increments) were examined adjusted for confounders and wear then, if significant, examined using isotemporal substitution modelling.

**Results:**

Waist circumference and BMI were significantly (p<0.05) associated with more prolonged sedentary time and less light-intensity activity. Light intensity activity was also significantly associated with lower fasting plasma glucose (relative rate: 0.98, 95% CI: 0.97, 1.00; p<0.05). No biomarker was significantly associated with non-prolonged sedentary time or MVPA. Lower mean prolonged sedentary time (−30 min/day) with higher mean light intensity time (+30 min/day) was significantly associated with lower waist circumference (β = −0.77, 95% CI: −1.33, −0.22 cm). Lower mean prolonged sedentary time (−30 min/day) with either 30 min/day higher mean non-prolonged sedentary time (β = −0.35, 95%CI: −0.70, −0.01 kg/m^2^) or light-intensity time (β = −0.36, −0.61, −0.11 kg/m^2^) was associated with significantly lower average BMI.

**Conclusions:**

Significantly improved mean levels of waist circumference and BMI were observed when shifting time from prolonged sedentary to non-prolonged sedentary or light-intensity activity (cross-sectionally). Lifestyle interventions in overweight/obese adults with type 2 diabetes might consider targeting shifts in these non-MVPA activities to more rigorously evaluate their potential cardiometabolic benefit in this population.

## Introduction

Regular participation in moderate- to vigorous-intensity physical activity (MVPA) is considered a key strategy in the prevention and management of type 2 diabetes mellitus [1]. Although successful exercise interventions have significantly improved glycaemic control in adults with this condition [2], increasing MVPA in this population presents challenges, with a meta-analysis showing interventions typically achieve weak or moderate effects [3]. It may therefore be useful to also explore high-volume, low-intensity activities that constitute the remaining waking hours, especially considering that adults with type 2 diabetes spend less than 5% of the day engaged in MVPA [4,5].

There is now rapidly emerging evidence, including in adults with type 2 diabetes [4,6], on the associations of sedentary time (adverse) and light-intensity activity (beneficial) on biomarkers of health and health outcomes [7–12]. These biomarkers and health outcomes include waist circumference, body mass index (BMI), high-density lipoprotein (HDL)-cholesterol, C-reactive protein, triacylglycerols, insulin, mortality, and depression [7–12]. In addition, there is emerging evidence on the potential benefits of regularly interrupting sedentary time [8,13,14] (i.e., reducing prolonged, unbroken sedentary time). Typically, these associations are examined adjusted for confounders only. Occasionally, associations are tested independently of (i.e., statistically adjusted for) all or some activities. Few studies (and none in adults with type 2 diabetes) have examined these associations while treating the activities as interdependent, i.e., that total hours in a day are finite so increasing time spent at any one activity level invariably decreases the remaining time spent at other levels. The isotemporal substitution approach [15] takes into account this interdependency by estimating the association with mean outcomes (e.g. mean biomarker levels) of increasing mean time spent in one activity (e.g. MVPA) by reducing mean time spent in another activity (e.g. sedentary) by an equivalent amount, keeping total time fixed. The collective impact from both increased MVPA and reduced sedentary (reallocating time from sedentary to MVPA) should logically be larger than when considering only one activity at a time, or adjusting for other activities.

Isotemporal substitution analysis, used widely in nutritional epidemiology, has recently been applied to physical activity to explore the relationships of various types and intensities of activity with several outcomes, including depression [16] and weight change in women [19]; older adults’ quality of life [17] and physical health and well-being [18]; and, cardio-metabolic biomarkers in the general adult population [19]. The latter two studies used device-based measures of activity (accelerometers) [18,19], thus enabling insights into the activity-health relationship across the activity spectrum (from sedentary through to MVPA). For example, Buman and colleagues [19] found that cross-sectional associations with insulin and triacylglycerols were equally strong for two plausible scenarios: decreasing mean sedentary time by 3 hours/day via additional time spent in light-intensity activity; and, decreasing mean sedentary time by 30 min/day via equivalently increasing mean MVPA. Examining various isotemporal substitutions may provide valuable insights into useful behavioural strategies for adults with type 2 diabetes, many of whom find participation in MVPA a considerable challenge [20].

Utilizing data from adults with type 2 diabetes with an overweight or obese BMI (≥25 kg/m^2^), the aim of this current study was to examine the associations of objectively-derived activity with biomarkers of cardiometabolic risk, using an isotemporal substitution approach. Time spent in each activity across the spectrum was assessed: the most active being MVPA, then light-intensity activity, and sedentary time occurring in short (<30 minute) and prolonged, unbroken (≥30 minute) bouts.

## Materials and Methods

### Study design

Living Well with Diabetes (LWWD) was a randomized controlled trial evaluating an 18-month (with follow-up at 24-months) telephone-delivered behavioural weight loss intervention versus usual care in adults with type 2 diabetes. Assessments were conducted at baseline, 6-, 12-, 18-, and 24-months; this study reports on the cross-sectional baseline data only (2009–2011). Baseline data were collected before randomization. Ethical approval was granted from The University of Queensland Behavioural and Social Sciences Ethical Review Committee (#2008000188; approved 26/02/2008). Detailed methods [21], and the primary outcome findings from the LWWD trial have been published [22,23]. The trial was registered with the Australian New Zealand Clinical Trials Register (www.anzctr.org.au: #ACTRN12608000203358).

### Participants

Participants were recruited from general practices from a large ethnically and socio-economically diverse community in Queensland, Australia. Key eligibility criteria were diagnosed type 2 diabetes, aged 20–75 years, BMI in the overweight or obese category (≥ 25 kg/m^2^) and/or inactive (not achieving ≥30 minutes of MVPA on five or more days/week), and without contradictions to an unsupervised physical activity and weight loss intervention [24]. In total, 302 participants provided informed consent (written and/or oral) and underwent baseline assessment [22]. Only those who met the BMI threshold for overweight (≥ 25 kg/m^2^) were included in the present study (n = 285).

### Data collection

Data were collected via objective measurements, telephone interviews, and self-administered questionnaires. Home visits were conducted by registered nurses and included anthropometric and fasting (minimum 10 hours) blood measures, which were sent to Sullivan Nicolaides Pathology for processing. Accelerometers, given to participants during the nurse visit to wear for seven days, were used to derive physical activity and sedentary time.

### Measures

#### Physical activity and sedentary time

Nurses fitted each participant with a dual-axis GT1M accelerometer (Actigraph, LLC, Fort Walton Beach, Florida), fastening the monitors firmly around the waist by elasticized bands positioned on the right mid axillary line. Monitors were set to record accelerometer counts in one minute epochs (Actilife V3.7), representing total vertical acceleration occurring over each minute. Participants were asked to wear the monitor during all waking hours (except during water-based activities) continuously for seven days, and to record time worn in a log. Monitor non-wear periods were excluded. These were identified by research staff who compared the accelerometer output with participants’ wearing logs to determine the precise times (approximately coinciding with participants’ self-reported wear/removal periods) that movement ceased or began. Using SAS 9.2, count per minute thresholds [25,26] were applied to calculate daily MVPA (≥1952), light-intensity activity (100 to 1951), and sedentary (<100) time, with prolonged sedentary time (sedentary time accumulated in bouts of 30 minutes or more) and non-prolonged sedentary time (sedentary time accumulated in bouts <30 minutes) calculated separately. Total time was calculated as the sum of time spent in all activities (prolonged sedentary, non-prolonged sedentary, light and MVPA). Data are reported as averages for valid days (days with > 10 hours wear and no minutes with counts ≥ 20,000), and are examined in units of 30 minutes per day (regression models) as well as percentages of wear time (descriptive analyses). Accelerometer compliance was high, with almost all participants (297/302) providing at least four valid days of data (72% had seven days).

#### Cardiometabolic outcomes

Weight (nearest 0.1kg), height (nearest 0.1cm), and waist circumference (nearest 0.5cm) were measured without shoes or heavy clothing using standard protocols [21]. The height and weight measures were used to calculate BMI (kg/m^2^). Glycated haemoglobin (HbA_1c_) was measured from whole blood samples by the high performance liquid chromatography method (ion-exchange with UV detection; Bio-Rad Variant II Turbo, Hemoglobin A_1c_ Program Reorder Pack, (270–2415)). The method for measuring plasma glucose was enzymatic reference with hexokinase [27]. HDL-cholesterol and triacylglycerols were measured by an enzymatic colorimetric assay with Roche Modular Chemistry Analyzer (Roche HDL-C Plus Reagent 1 Cat No. 04713311; Roche Triglyceride Reagent Cat No. L1876040).

#### Potential confounding variables

Telephone interviews collected self-report data on: demographics (gender, age, education, employment, income, country of birth and ethnicity), diet, smoking status, use of weight loss aids, chronic physical and psychological conditions, and diabetes history and management. Dietary intake was measured using a validated food frequency questionnaire that assessed food intake over the previous month (Anti-Cancer Council of Victoria Food Frequency Questionnaire version 2) [28]. Average daily energy intake was then calculated using the NUTTAB95 nutrient composition database [29]. The diet quality index-revised score was used to calculate dietary quality [30]. Use of diabetes medications (insulin, traditional oral and glucagon-like-peptide-1 agents) and any use (yes/no) of antihypertensive and lipid-lowering medications were collected during the nurse visit.

### Data Analysis

Analyses were conducted using SPSS version 20.0 (IBM Corp). Significance was set at p<0.05, two-tailed or p<0.01 for interactions (in view of the multiple hypothesis testing). The present analysis examines all participants with an overweight/obese BMI with data on relevant variables, including at least four days of accelerometer data (n = 279). As all interactions (by age, gender, and insulin use) were not statistically significant, pooled analyses are presented.

Associations with biomarkers were examined based on a series of linear regression models, with results presented as unstandardized regression coefficients (for waist circumference, BMI, HDL-cholesterol, blood pressure) or, back-transformed as relative rates for log transformed outcomes (HbA_1c_, fasting plasma glucose, triacylglycerols), per 30 min/day of activity. Models did not display non-normality or heteroscedasticity; log transformation was required at times to achieve this. Minimum differences of interest [21] were: 5cm waist circumference; 1.7 kg/m^2^ BMI; 5% (i.e., 0.06 mM) HDL-cholesterol; 5mmHg systolic blood pressure; 3mmHg diastolic blood pressure; and, 10% for relative rates per 2.5 hours/day for sedentary and light-intensity activity or per 1 hour/day for MVPA. All regression models were adjusted for confounders (age, and socio-demographic, behavioural, and medical variables that were selected via backwards elimination as significantly associated with the outcome at p<0.2, as listed in S1 Table).

Three models are reported. Model 1, the model traditionally presented in physical activity research, examined the associations of each activity separately adjusted for wear time and confounders. Model 2 examined the associations for each activity adjusted for every other activity as well as confounders (S2 Table; Model 2). Here, all variance inflation factors were < 2, and were not indicative of multicollinearity. Finally, for all biomarkers that had been significantly associated with any of the activities from Model 1, an isotemporal substitution model was used (Model 3) to examine the time substitution effects of one activity for another [15]. Model 3 adjusted for confounders, total wear time, and time spent in each activity (except the activity for which the mean time is being reduced). The regression coefficients for the included activities (all in 30 min/day units) in this model represent the estimated shift in the mean biomarker outcome of increasing the mean time in these activities by 30 min/day while reducing the mean time in the omitted activity by 30 min/day, keeping total wear time fixed (isotemporal). For log-transformed outcomes, the coefficients are reported as relative rates. Notably, these are cross-sectional associations, not causal associations of individuals replacing time in one activity with another.

## Results

The socio-demographic and cardio-metabolic characteristics of participants are detailed in Table 1. Representativeness of the LWWD sample has previously been reported [22]. The majority of participants (158 men; 121 women) were Australian born, Caucasian, and married. The median duration of their diabetes was five years with a median HbA_1c_ level of 7.1%. Most participants (77%) used traditional oral medications, 14% used insulin, and most had at least one other chronic condition (primarily cardiovascular related). Participants’ BMI was mostly in the obese category (71.7%, BMI ≥30 kg/m^2^), with 32 participants (11.5%) with a BMI of ≥40 kg/m^2^. The majority of observed waking hours were spent sedentary (62.7 [SD 10.8]%), with the rest of time disproportionately distributed between light-intensity activities (35.0 [SD 10.0]%) and MVPA (2.2 [SD 2.1]%). Over one quarter (25.4%) of all sedentary time was accrued in prolonged bouts of ≥30 minutes (18.5 [SD 11.1]% of total time).

**Table 1:** Socio-demographic, clinical, and accelerometer derived activity characteristics of participants (n = 279).

**Characteristic**	Summary ^a^
Age, years	58.2 (8.6)
Men, %	56.6 (158)
Duration of diabetes, years, *median (25* ^*th*^, *75* ^*th*^ *percentile)*	5 (2, 8)
< High school education, %	11.8 (33)
Born in Australia, %	70.3 (196)
Caucasian, %	90.0 (251)
Retired, %	28.3 (79)
Household income <$1000/week, %	7.9 (22)
Married, %	81.4 (227)
Use traditional oral hypoglycaemics, %	77.1 (215)
Use insulin, %	14.0 (39)
Use glucagon-like-peptide-1 agents (incretins), %	3.9 (11)
Diagnosed CVD-related condition, %	81.4 (227)
Diagnosed musculoskeletal condition, %	34.1 (95)
Diagnosed lung condition, %	10.4 (29)
Diagnosed depression and/or anxiety, %	16.5 (46)
Smoking status: never smoker, %	49.1 (137)
Smoking status: ex-smoker, %	41.2 (115)
Smoking status: current smoker, %	9.7 (27)
No use of weight loss aids in the past 6 months, %	78.9 (220)
Total energy intake, MJ	7.0 (2.2)
Diet quality index-revised score (0–100)	65.4 (11.1)
Waist circumference, cm	110.8 (12.6)
Body mass index (BMI), kg/m^2^	33.6 (5.5)
Glycated haemoglobin (HbA_1c_), %, *median (25* ^*th*^, *75* ^*th*^ *percentile)*	7.1 (6.4, 8.0)
Fasting blood glucose, mM, *median (25* ^*th*^, *75* ^*th*^ *percentile)*	7.2 (6.3, 9.2)
Triacylglycerols, mM *median (25* ^*th*^, *75* ^*th*^ *percentile)*	1.6 (1.2, 2.2)
High-density-lipoprotein (HDL)-cholesterol, mM	1.16 (0.29)
Systolic blood pressure, mmHg ^b^	134.0 (14.1)
Diastolic blood pressure, mmHg ^b^	82.1 (8.8)
Accelerometer wear time, min/day ^b^	811.2 (99.3)
Moderate to vigorous intensity physical activity, min/day ^b^	17.9 (16.6)
Light intensity activity, min/day ^b^	282.7 (81.8)
Non-prolonged sedentary time (in ≥30 minute bouts), min/day ^b^	359.6 (74.2)
Prolonged sedentary time (in ≥30 minutes bouts), min/day ^b^	151.0 (92.7)

^a^ Data are mean (SD), % (n) or median (25^th^, 75^th^ percentile).

^b^ ActiGraph GT1M accelerometer data, 60-second epochs, count per minute thresholds applied (<100 = sedentary/ 100 to <1952 = light / ≥1952 = moderate to vigorous), not adjusted for accelerometer wear time.

### Associations of activity outcomes with cardio-metabolic outcomes

Table 2 shows the associations of each activity with cardio-metabolic biomarkers from Model 1. Adjusted for wear and confounders, prolonged sedentary time had significant associations with higher waist circumference and BMI (≈0.67 cm and 0.33 kg/m^2^ per 30 min/day respectively). No significant associations of prolonged sedentary time were observed with the other biomarkers, however the 95% confidence intervals could not rule out meaningful associations with glucose and triacylglycerols (i.e., the findings were inconclusive). Statistically significant associations of light-intensity activity were observed with lower waist circumference, BMI, and fasting plasma glucose (≈0.61 cm, 0.29 kg/m^2^ and 2% lower per 30 min/day respectively). Findings were inconclusive for HDL-cholesterol. Non-prolonged sedentary time and MVPA did not have significant associations with any outcome, though most of these findings were inconclusive. None of these associations remained statistically significant after adjustment for other activities (Model 2; S2 Table).

**Table 2:** Cross-sectional associations (Model 1) of each 30 minutes per day of prolonged sedentary, non-prolonged sedentary, light intensity activity and moderate to vigorous intensity activity (MVPA) with continuous cardio-metabolic biomarkers in n = 279 overweight/obese adults with type 2 diabetes.

**Outcome variable**	**Regression coefficients (β) or relative rate (RR) and 95% Confidence Interval**			
	**Prolonged sedentary** ^a^	**Non-prolonged sedentary** ^a^	**Light intensity** ^a^	**MVPA** ^a^
	**(per 30 min/day)**	**(per 30 min/day)**	**(per 30 min/day)**	**(per 30 min/day)**
Waist circumference, *cm* (β)	**0.67 (0.02, 1.14)** **	−0.43 (−1.19, 0.32)	−**0.61 (**−**1.14,** −**0.09)** *	0.03 (−2.51, 2.56)
Body mass index, *kg/m* ^*2*^ (β)	**0.33 (0.12, 0.54)** **	−0.24 (−0.57, 0.09)	−**0.29 (**−**0.52,** −**0.05)** *	−0.01 (-1.10, 1.09)
HbA_1c_, % (RR)	1.01 (1.00, 1.01)	1.00 (0.99, 1.01)	0.99 (0.99, 1.00)	0.98 (0.94, 1.01)
Fasting plasma glucose, *mM* (RR)	1.01 (1.00, 1.02)	1.01 (0.99, 1.03)	**0.98 (0.97, 1.00)** *	0.94 (0.88, 1.00)
Triacylglycerols, *mM* (RR)	1.01 (0.99, 1.03)	1.01 (0.98, 1.04)	0.99 (0.97, 1.01)	0.97 (0.88, 1.06)
HDL-cholesterol, *mM* (β)	0.00 (−0.01, 0.01)	−0.01 (−0.02, 0.01)	0.01 (0.00, 0.02)	−0.01 (−0.06, 0.05)
Systolic blood pressure, *mmHg* (β)	0.32 (−0.23, 0.88)	−0.56 (−1.42, 0.30)	−0.17 (−0.79, 0.45)	1.31 (−1.51, 4.13)
Diastolic blood pressure, *mmHg* (β)	0.11 (−0.24, 0.45)	−0.53 (−1.08, 0.02)	0.07 (−0.31, 0.46)	1.19 (−0.63, 3.00)

* *p*<0.05;

** *p*<0.01;

*** *p*<0.001.

^a^ Activities as estimated from ActiGraph GT1M accelerometers, 60-second epoch, count per minute thresholds: <100 (sedentary); 100 to <1952 (light); ≥ 1952 (moderate to vigorous physical activity; MVPA), with sedentary time in bouts ≥30 minutes classed as prolonged and <30 minutes classed as non-prolonged.

Data presented as unstandardized regression coefficients (β) or relative rates (RR) with 95% confidence intervals.

RR >1 indicates an increase while RR <1 indicates a decrease in mean cardiometabolic biomarker levels per additional 30 mins/day of activity. All models adjusted for age and other potential confounders (listed in S1 Table) and total wear time (Model 1). Minimum differences of interest per 2.5 hours/day for sedentary and light-intensity activity or per 1 hour/day for MVPA were: 5cm waist circumference; 1.7 kg/m^2^ BMI; 5% (i.e., 0.06 mM) HDL-cholesterol; 5mmHg systolic blood pressure; 3mmHg diastolic blood pressure; and, 10% for all relative rates.

Table 3 shows the isotemporal substitution results (Model 3) for those biomarkers significantly associated with any activity in Model 1 (waist circumference, BMI and fasting plasma glucose). Cross-sectionally, lower mean prolonged sedentary time (−30 min/day) when coupled with higher mean light-intensity activity time (+30 min/day) was associated with significantly lower mean waist circumference (≈0.77 cm). Significant associations with BMI were observed with lower mean prolonged sedentary time (-30 min/day) coupled with either higher mean non-prolonged sedentary time or light-intensity time (+30 min/day), (≈0.35 kg/m^2^ and 0.36 kg/m^2^, equivalent to a meaningful amount per 2.5 hours/day of 1.75 kg/m^2^ and 1.80 kg/m^2^ respectively). Other reallocations for waist circumference and BMI were not statistically significant, however, clinically meaningful associations could not be ruled out (with the exception of the association with waist circumference of reallocating non-prolonged sedentary time to light-intensity activity). Similarly, although no statistically significant associations were observed for any of the reallocations for fasting plasma glucose, the confidence intervals did not preclude clinically meaningful associations.

**Table 3:** Cross-sectional associations (Model 3) with waist circumference, BMI and fasting blood glucose in adults with type 2 diabetes and an overweight or obese BMI (n = 279) that were estimated for 30 minutes per day lower mean time spent in sedentary and light activity categories coupled with equivalent 30 minutes per day higher mean time spent in other activity categories.

**Mean time in activity is 30 min/day …**		**Biomarker outcome**		
**… lower**	**… higher**	**Waist circumference, cm**	**Body mass index, kg/m^2^**	**Fasting blood glucose (log) mM**
		**β (95% CI)**	**β (95% CI)**	**RR (95% CI)**
Prolonged sedentary ^a^	Non-prolonged sedentary ^a^	−0.69 (−1.46, 0.08)	−**0.35 (**−**0.70,** −**0.01)** *	1.01 (0.99, 1.03)
	Light activity ^a^	−**0.77 (**−**1.33,** −**0.22)** **	−**0.36 (**−**0.61,** −**0.11)** **	0.99 (0.97, 1.00)
	MVPA ^a^	0.64 (−1.96, 3.24)	0.20 (−0.93, 1.32)	0.96 (0.90, 1.03)
Non-prolonged sedentary ^a^	Light activity ^a^	−0.08 (−0.93, 0.76)	−0.01 (−0.38, 0.37)	0.98 (0.96, 1.00)
	MVPA ^a^	1.33 (−1.31, 3.96)	0.55 (−0.58, 1.68)	0.96 (0.89, 1.02)
Light activity ^a^	MVPA ^a^	1.41 (−1.37, 4.19)	0.56 (−0.64, 1.76)	0.98 (0.91, 1.05)

* *p*<0.05;

** *p*<0.01;

*** *p*<0.001

^a^ Activities as estimated from ActiGraph GT1M accelerometers, 60-second epoch, count per minute thresholds: <100 (sedentary); 100 to <1952 (light); ≥ 1952 (moderate to vigorous physical activity; MVPA), with sedentary time in bouts ≥30 minutes classed as prolonged and <30 minutes classed as non-prolonged.

Data presented are unstandardized regression coefficients (β) or relative rates (RR) for log-transformed outcomes with 95% confidence interval (CI), from models that include age, other potential confounders (listed in S1 Table), total wear time and all activities for which mean time is being increased (Model 3). RR >1 indicates an increase while RR <1 indicates a decrease in mean cardiometabolic biomarkers per 30 min/day reallocated from one activity to another.

## Discussion

This study examined the associations of accelerometer-derived activity across the intensity spectrum—from sedentary through to MVPA—with biomarkers of cardiovascular risk in a clinical population of adults with type 2 diabetes and with an overweight/obese BMI. Consistent with accelerometer measurement studies in both the general population [31] and in adults with type 2 diabetes [5], the majority of waking hours were spent sedentary (63%), with very little time spent in MVPA (2%). Statistically significant associations of prolonged sedentary time and light-intensity activity with cardio-metabolic biomarkers were observed, particularly with markers of body composition. Specifically, higher mean waist circumference (an indicator of central obesity) and BMI (a crude indicator of general obesity) were observed with more prolonged sedentary time and less light-intensity activity. More light-intensity activity was also associated with lower fasting plasma glucose.

An important element of this study was the differentiation of sedentary time occurring in prolonged, unbroken bouts, from other shorter bouts. Prolonged sedentary time was common, constituting 25% of total sedentary time and ≈19% of all worn waking hours. Although the cut-point for prolonged sedentary time (≥30 minutes) is somewhat arbitrary, this distinction between accumulation patterns acknowledges that all forms of sedentary time are not necessarily equally detrimental. For example, it is less likely that sitting for one to two minutes will have harmful effects compared to sitting for longer periods continuously. Here, bed-rest studies [32], alongside more recent experimental studies using a seated posture [13,14], have highlighted the acute, detrimental effects of prolonged, unbroken bouts of sitting or lying: a finding supported by the cross-sectional associations observed here.

The cross-sectional associations observed in this study, adjusted for wear time and confounders, were largely consistent with previous research in adults with type 2 diabetes [4,6]. A novel element of this study was that the activity-health associations were also examined with consideration that increasing time spent in one activity necessarily displaces time spent in another. For waist circumference and BMI, the magnitude of the associations modelled using this isotemporal substitution method (Model 3) were stronger than for each activity considered separately (Model 1) or adjusted for other activities (Model 2), as is typically done in cross-sectional accelerometer studies of activity-biomarker relationships. Notably, reducing mean prolonged sedentary time while increasing mean light-intensity activity had meaningful, significant associations with lower average waist circumference and BMI, while reducing mean prolonged sedentary time while increasing mean non-prolonged sedentary time was associated with significantly lower BMI. As such, these cross-sectional findings suggest that even an activity goal as modest as reducing prolonged sedentary time by regularly breaking it up should be investigated in intervention studies as a strategy to improve body composition for overweight/obese adults with type 2 diabetes. Indeed, a recent sedentary behaviour intervention targeting reductions in prolonged sedentary time in a general adult population saw a significant, beneficial intervention effects on waist circumference, despite relatively modest activity changes [33].

Key strengths of the study include the objective activity measures examined across multiple intensities and the focus on a clinically important population (i.e., adults with an overweight/obese BMI with type 2 diabetes). Limitations include the cross-sectional design and the lack of an objective assessment of sleep. Additionally, this secondary analysis was not powered *a priori* for these research questions, and most confidence intervals around the null associations did not exclude meaningful benefit and/or harm as unlikely. Further, many hypotheses were tested without adjustment of significance (due to type II error concerns); the possibility exists that significant findings may be spurious. In particular, the non-significant associations of MVPA with outcomes should not be interpreted as MVPA being unimportant for cardio-metabolic health. Here, it is possible that associations were missed due to insufficient sample size, measurement error associated with accelerometer-derived activity classification [34], and/or the very low levels of MVPA in this study population (≈2%). Indeed, findings using the isotemporal substitution approach in a general US adult population found MVPA to be the most potent health-enhancing, time-dependent behaviour [19]. Although widely used accelerometer cut-points were utilized to differentiate the activity intensities, all cut-points inherently have some misclassification [34]. Further, the activity monitor used primarily detects ambulatory activity, with activity classifications based on estimated energy expenditure, not posture. Thus, misclassification is likely, especially for light-intensity activities involving little vertical movement (such as standing still) as they could be misclassified as non-prolonged sedentary time. The apparent benefit of reallocating time from prolonged sedentary to non-prolonged sedentary time could reflect the importance of accumulation patterns, or of behaviours such as standing. Future studies should consider using postural sensors, and/or alternative analytic techniques [35] to reduce such misclassification. Future research could also assess the potential benefits/harms of reallocating time across the 24-hour day between different waking activities and sleep.

In conclusion, this study found high levels of prolonged, unbroken sedentary time in overweight/obese adults with type 2 diabetes. Reductions in prolonged sedentary time, even without corresponding increases in MVPA, showed associations (cross-sectionally) with lower BMI and waist circumference. These findings, in conjunction with the limited success of interventions aimed at increasing MVPA in this population, suggest that high-volume, low-intensity strategies for activity change should also be investigated by intervention studies for their feasibility and potential benefit on body composition and other cardio-metabolic biomarkers in adults with type 2 diabetes.

## Supporting Information

S1 Table List of confounders specific to each outcome variable. (DOCX)

S2 Table Associations of activities ^a^ (Model 2) with continuous cardio-metabolic biomarkers, adjusted for all other activities and potential confounders, in n = 279 adults with type 2 diabetes and an overweight/obese BMI classification. (DOCX)
