# Supplementary material for: Accelerometer-Derived Sedentary and Physical Activity Time in Overweight/Obese Adults with Type 2 Diabetes: Cross-Sectional Associations with Cardiometabolic Biomarkers
Source: PLoS One. 2015 Mar 16;10(3):e0119140. doi: 10.1371/journal.pone.0119140 (PMC4361561; doi:10.1371/journal.pone.0119140)
Supplement: S1 Table — (DOCX) [file pone.0119140.s001.docx]

**Table S1**: List of confounders specific to each outcome variable

| **Outcome variable** | **Confounders for all models of the outcome variable** ^a^ |
| --- | --- |
| Waist circumference, *cm* | age (years), diet quality index-revised score, duration of diabetes (quartiles), gender (male/female), traditional oral hypoglycaemic use (yes/no), musculoskeletal condition (yes/no), depression and/or anxiety (yes/no), employment (retired: yes/no), weight loss aids in last 6 months (yes/no), born in Australia (yes/no) |
| Body mass index, *kg/m^2^* | diet quality index-revised score, musculoskeletal condition (yes/no), weight loss aids in last 6 months (yes/no), Caucasian (yes/no) |
| HbA_1c_ (log transformed), *%* | energy intake (kJ), diet quality index-revised score (0-100), duration of diabetes (quartiles), traditional oral hypoglycaemic use (yes/no), use of insulin (yes/no), depression and/or anxiety (yes/no), Caucasian (yes/no) |
| Fasting plasma glucose (log), *mM* | energy intake (kJ), gender (male/female), traditional oral hypoglycaemic use (yes/no), Caucasian (yes/no), married (yes/no) |
| Triacylglycerols (log), *mM* | diet quality index-revised score, traditional oral hypoglycaemic use (yes/no), use of glucagon-like-peptide-1 agents (incretins; yes/no), CVD-related condition (yes/no), born in Australia (yes/no) |
| HDL-cholesterol, *mM* | age (years), born in Australia (yes/no), gender (male/female), traditional oral hypoglycaemic use (yes/no), CVD-related condition (yes/no) |
| Systolic blood pressure, *mmHg* | age (years), diet quality index-revised score (0-100), CVD-related condition (yes/no), employment (retired: yes/no), Caucasian (yes/no) |
| Diastolic blood pressure, *mmHg* | age (years), duration of diabetes (quartiles), CVD-related condition (yes/no), smoking status (never- /ex- /current) |

^a^ Confounders for each outcome (listed above) were variables that were significant at p<0.2 in backward elimination models and age. The backward elimination for each outcome began with the following full list of variables: age (years), gender (male/female), BMI (kg/m^2^) or waist circumference (cm, for waist circumference models), log HbA1c (mM), use of insulin (yes/no), traditional oral hypoglycaemic use (yes/no), use of glucagon-like-peptide-1 agents (incretins; yes/no), diabetes duration (quartiles), household income (<$1000 per week: yes/ no or missing), education (< high school: yes/no), weight loss aids in last 6 months (yes/no), smoking status (never- /ex- /current-), CVD-related condition (CVD, stroke, high cholesterol or hypertension: yes/no), musculoskeletal condition (yes/no), depression and/or anxiety (yes/no), employment (retired: yes/no), born in Australia (yes/no), Caucasian (yes/no), energy intake (kJ), diet quality index-revised score (0-100)
