# Supplementary material for: Accelerometer-Derived Sedentary and Physical Activity Time in Overweight/Obese Adults with Type 2 Diabetes: Cross-Sectional Associations with Cardiometabolic Biomarkers
Source: PLoS One. 2015 Mar 16;10(3):e0119140. doi: 10.1371/journal.pone.0119140 (PMC4361561; doi:10.1371/journal.pone.0119140)
Supplement: S2 Table — (DOCX) [file pone.0119140.s002.docx]

**Table S2**: Associations of activities **^a^** (Model 2) with continuous cardio-metabolic biomarkers, adjusted for all other activities and potential confounders, in n=279 adults with type 2 diabetes and an overweight/obese BMI classification

| **Outcome variable** | **Regression coefficients (β) or relative rate (RR) and 95% confidence interval** | | | |
| --- | --- | --- | --- | --- |
|  | **Prolonged sedentary** | **Non-prolonged sedentary** | **Light intensity** | **MVPA** |
|  | **(30 min/day)** | **(30 min/day)** | **(30 min/day)** | **(30 min/day)** |
| Waist circumference, *cm* (β) | 0.24 (-0.35, 0.83) | -0.45 (-1.01, 0.10) | -0.54 (-1.20, 0.13) | 0.87 (-1.77, 3.51) |
| Body mass index, *kg/m^2^* (β) | 0.17 (-0.09, 0.43) | -0.18 (-0.43, 0.06) | -0.19 (-0.49, 0.10) | 0.37 (-0.77, 1.50) |
| HbA_1c_, *%* (RR) | **1.01 (1.00, 1.02)*** | 1.01 (1.00, 1.02) | 1.00 (0.99, 1.01) | 0.99 (0.96, 1.03) |
| Fasting plasma glucose, *mM* (RR) | 1.01 (0.99, 1.02) | 1.01 (1.00, 1.03) | 0.99 (0.98, 1.01) | 0.97 (0.91, 1.04) |
| Triacylglycerols, *mM* (RR) | 1.01 (0.98, 1.03) | 1.01 (0.99, 1.04) | 1.00 (0.97, 1.02) | 0.99 (0.89, 1.10) |
| HDL-cholesterol, *mM* (β) | 0.00 (-0.01, 0.02) | 0.00 (-0.01, 0.01) | 0.01 (0.00, 0.03) | -0.02 (-0.08, 0.04) |
| Systolic blood pressure, *mmHg* (β) | 0.13 (-0.56, 0.82) | -0.48 (-1.13, 0.17) | -0.24 (-1.01, 0.54) | 1.47 (-1.49, 4.43) |
| Diastolic blood pressure, *mmHg* (β) | 0.24 (-0.20, 0.69) | -0.25 (-0.66, 0.16) | 0.18 (-0.31, 0.68) | 1.19 (-0.72, 3.09) |

***** *p*<0.05; ** *p*<0.01; *** *p*<0.001

^a^ Activities as estimated from ActiGraph GT1M accelerometers, 60-second epoch, count per minute thresholds: <100 (sedentary); 100 to <1952 (light); ≥ 1952 (moderate to vigorous physical activity; MVPA), with sedentary time in bouts ≥30 minutes classed as prolonged and <30 minutes classed as non-prolonged.

Data presented as unstandardized regression coefficients (β) or relative rates (RR) with 95% confidence intervals. Models adjusted for age, other potential confounders (listed in Supplementary Table 1) and for all other activities (Model 2). RR >1 indicates an increase while RR<1 indicates a decrease in mean cardiometabolic biomarkers per 30 min/day of activity. Variance inflation factors were <2 for all models.
